# Peripheral Lymphocyte Subsets Predict Efficacy and Overall Survival in Gastric Cancer Patients Receiving Preoperative Chemotherapy: A Retrospective Cohort Study

**DOI:** 10.1002/cnr2.70704

**Published:** 2026-10-13

**Authors:** Zirui Xue, Dan Liu, Zhaodong Sun, Bosen Li, Xinyou Liu, Junjie Zhao, Haojie Li, Xuefei Wang, Zhaoming Wang

**Affiliations:** ^1^ Department of Gastrointestinal Surgery Zhongshan Hospital, Fudan University Shanghai China; ^2^ Gastric Cancer Center Zhongshan Hospital, Fudan University Shanghai China; ^3^ Department of General Surgery Zhongshan Hospital (Xiamen), Fudan University Xiamen China; ^4^ Xiamen Clinical Research Center for Cancer Therapy Xiamen China

**Keywords:** gastric cancer, pathological complete response, peripheral lymphocyte subset, systemic immunity

## Abstract

**Background:**

Pathologic complete response (pCR), which reflects the local immune status and preoperative chemotherapy efficacy, has garnered significant attention in gastric cancer research. However, the predictive value of peripheral lymphocyte subsets for pCR in gastric cancer patients receiving preoperative chemotherapy remains unclear.

**Aims:**

This study aims to assess the correlation between preoperative peripheral lymphocyte subsets and pCR in gastric cancer patients.

**Methods and Results:**

In this retrospective study, gastric cancer patients who underwent curative gastrectomy following preoperative chemotherapy were included. Cytokines and peripheral lymphocytes were measured using flow cytometry. Clinicopathological characteristics, peripheral lymphocyte data and cytokine data were collected for the analysis. Overall survival (OS) was analyzed using Kaplan–Meier curves. Of 173 patients, 15 (8.7%) achieved pCR. A lower proportion of CD19^+^ B cells (7.5% vs. 10.4%, *p* = 0.008) was associated with pCR, and a high absolute count of NK cells was associated with better OS (*p* = 0.044).

**Conclusions:**

Our study indicated that a lower proportion of CD19^+^ B cells may serve as a predictive biomarker for pCR, while an increased peripheral NK cell count could function as an auxiliary factor for risk stratification when combined with clinical parameters to assess long‐term outcomes in gastric cancer patients receiving preoperative chemotherapy.

## 1 Introduction

Gastric cancer is the fifth most frequently diagnosed cancer and the fifth leading cause of cancer death worldwide [1, 2]. Due to the absence of typical symptoms and screening systems for early gastric cancer, about 70% of gastric cancer patients are diagnosed with locally advanced disease. Preoperative treatments, such as neoadjuvant and conversion therapies, are commonly used to downstage tumors, improve R0 resection rates, and increase the chance of curative surgery [3, 4]. Despite advances in comprehensive treatment, the postoperative recurrence rate remains high, leading to poor prognosis. Identifying risk factors and predictive markers associated with poor outcomes, along with implementing early interventions and adjusting treatment plans, is of critical importance.

Pathological complete response (pCR), which is closely linked to disease‐free survival (DFS) and overall survival (OS) in breast cancer and colorectal cancer [5, 6], indicates the total elimination of cancer cells by preoperative treatments and shows potential as a surrogate marker for long‐term survival [7]. Our previous study showed that systemic immune activity significantly impacts the effectiveness of therapeutic interventions in gastric cancer. Additionally, pCR patients demonstrated an improved OS, which was reflected in peripheral immune markers [8].

Currently, the tumor immunology research has focused heavily on local immune responses within the tumor microenvironment (TME) [9, 10, 11]. However, the overall activity of the immune system is also correlated with cancer prognosis, which inspired us to further investigate the relationship between systemic immune activity and the efficacy of preoperative chemotherapy. The impact of chemotherapy on the immune system is highly dependent on the integrity of immune function, emphasizing the need to investigate the association between global immune landscape and peripheral immune biomarkers. Peripheral lymphocytes, particularly vital for immune surveillance, patrol the bloodstream to detect and eliminate cancer cells [12]. Alterations in lymphocyte subsets have also been associated with cancer patient prognosis, including ovarian cancer [13], primary liver cancer [14], and breast cancer [15]. In gastric cancer, peripheral inflammation markers like neutrophil‐to‐lymphocyte ratio (NLR), platelet‐to‐lymphocyte ratio (PLR), systemic immune inflammation index (SII), and high peripheral lymphocyte count have been used to predict the effectiveness of preoperative chemotherapy and serve as significant prognostic biomarkers for poor OS [16, 17].

Platelets, lymphocytes, and neutrophils, which are easily detected in routine blood tests, play important roles in the immune response [18], inflammatory and anti‐inflammatory processes, and coagulation status related to tumor progression and prognosis in various solid tumors [19]. However, their predictive accuracy is limited, as they primarily reflect an underlying systemic pro‐inflammatory status without identifying specific mechanisms [20], and there is no uniform standard definition. However, the predictive value of peripheral lymphocyte subsets on pCR in gastric cancer patients receiving preoperative chemotherapy remains unknown. To address these questions, we conducted a retrospective monocentric analysis to explore the association between peripheral lymphocyte subsets and pCR in gastric cancer patients, as well as their correlation with OS.

## 2 Methods

### 2.1 Patients

One hundred and seventy‐three patients underwent gastric resection between June 2014 and May 2023 at Zhongshan Hospital, Fudan University. All patients received preoperative chemotherapy and curative‐intent resection with D2 lymph node dissection. Gastric adenocarcinoma was confirmed in all patients. Other inclusion criteria consisted of the age of patients and donors within the range of 18–75 years old. Exclusion criteria were as follows: other histology subtype including squamous cell carcinoma, neuroendocrine tumors, and stromal tumors; concurrent primary malignant disease of other organs; R1/R2 resection; preoperative chemotherapy combined with radiotherapy, immunotherapy target therapy, or HIPEC; unclear preoperative treatment regimen. The therapeutic response of patients was evaluated according to the RECIST 1.1 criteria.

### 2.2 Blood Parameter Assessment

Peripheral blood samples were collected following the initial round of preoperative chemotherapy to facilitate analysis. All laboratory parameters used in this study were derived from the electronic medical record system of the Department of Clinical Laboratory at Zhongshan Hospital, Fudan University. The lymphocyte detection was performed using the BD FACSLyric Flow Cytometry System (BD Biosciences, San Jose, CA, USA). The levels of TNF, sIL‐2R, interleukin‐6 (IL‐6), and interleukin‐8 (IL‐8) was tested by Bricyte E6 Flow cytometer (Mindray Bio‐Medical Electronics Co. Ltd. Shenzhen, China). The ratio of lymphocytes is defined as the proportion of lymphocytes in each group relative to the total peripheral blood lymphocytes, and the absolute lymphocyte counts are reported as cells per microliter (cells/μL) of peripheral blood. PBMCs were isolated from gastric cancer patients using Ficoll gradient centrifugation [21]. Peripheral lymphocyte subsets consisted of five items that were selected for detailed phenotypic analysis, using the following surface marker combinations: CD3^+^ T cells (CD45^+^ CD3^+^), CD8^+^ T cells (CD45^+^ CD3^+^ CD8^+^), CD4^+^ T cells (CD45^+^ CD3^+^ CD4^+^), CD19^+^ B cells (CD45^+^CD19^+^), CD16^+^CD56^+^ NK cells (CD3^−^CD16^+^ CD56^+^) (Figure S3).

### 2.3 Treatment Intent

Conversion therapy in gastric cancer is defined as the unresectable or borderline resectable tumors for surgical technical and/or oncological reasons; after active and effective chemotherapy and other comprehensive treatment, the primary gastric lesions can be reduced to a lower stage, while the metastatic lesions can be effectively controlled, to achieve R0 resection and improve the long‐term survival rate [22].

### 2.4 Clinical Response

Pathological complete response (pCR)‘ is defined as the absence of any remaining tumor component in both the primary tumor and the dissected regional lymph node [23]. In conversion therapy, pCR was defined as the absence of any residual tumor components in the primary site, lymph nodes, or metastatic lesions, with cytology clearance required for peritoneal metastasis. Overall survival (OS) is defined as the length of time from the date of diagnosis (pathology‐confirmed) to death of any cause or to the last follow‐up.

### 2.5 Public Data Assessment

For the Yonsei cohort, clinical characteristics such as sex, age, OS, TNM stage were included (https://www.ncbi.nlm.nih.gov/geo/query/acc.cgi?acc=GSE84437), 433 patients were selected from the GSE84437 dataset, excluding 50 patients due to missing survival data. The clinicopathologic information and transcriptomic data and of the Yonsei cohort were downloaded from Gene Expression Omnibus (https://www.ncbi.nlm.nih.gov/geo/) and the transcriptomic data was normalized by the formula log_2_(FPKM + 1).

The single‐cell sequencing data of gastric cancer from GSE206785 was used to construct NK cells (NCR1^+^, FGFBP2^+^) and B cells (CD19^+^, MS4A1^+^) gene set, high and low NK/B cell infiltration were defined by the median gene signature score [24].

### 2.6 Statistical Analysis

Categorical variables were analyzed using chi‐square test. Continuous variables were analyzed using *t*‐test and nonparametric numeric data were analyzed using Mann–Whitney rank sum test. Univariate analyses were performed using a logistic regression model. The odds ratio (OR) was reported with a 95% confidence interval (CI). Receiver operating characteristic (ROC) curve analysis were used to determine the optimal cut‐off levels and areas under the curves (AUCs) of peripheral lymphocyte parameters and their ratios. The cytokines correlation analysis was evaluated with Spearman's test. The Ef grade was applied to select the optimal cut‐off points. The Kaplan–Meier method and the Log‐rank test were used for survival analysis. *p* < 0.05 was considered statistically significant. Multivariate analysis of Cox regression model was applied to estimate hazard ratios (HRs) and 95% CIs. Statistical analyses were conducted by using GraphPad Prism (version 10.1.1), and R (version 4.3.2).

## 3 Results

### 3.1 The Baseline Characteristics of Included Patients

The study flowchart was presented in Figure 1. The baseline characteristics of all patients were given, 71.7% of whom were male (Table 1). Patients aged 60 years or older comprised 52.0% of the cohort. One hundred and sixty‐nine patients (97.7%) exhibited lymphatic invasion, and 57 patients (32.9%) had distant metastasis. Thirty‐five patients were diagnosed as signet ring cell carcinoma. Sixty‐five patients (37.6%) were diagnosed as intestinal type, 35 patients (20.2%) were diagnosed as diffuse type, and 35 patients (20.2%) were diagnosed as mixed type, according to Lauren classification. One hundred and five patients (60.5%) received neoadjuvant treatment, and 68 patients (39.3%) received conversion treatment. Among all patients, 158 patients (91.3%) were non‐pCR, and 15 patients (8.7%) achieved pCR. However, no statistically significant difference was observed between the pCR and non‐pCR patients (*p* = 0.600) (Figure S1).

**FIGURE 1:**
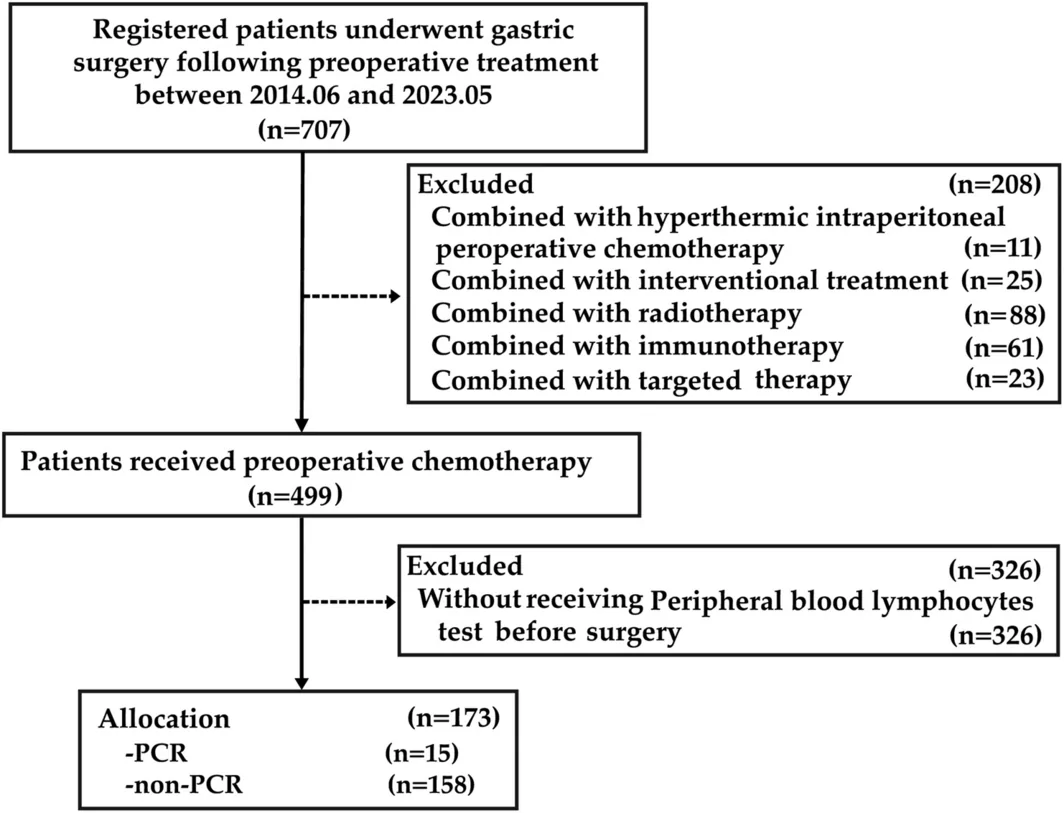
Flowchart of patient inclusion and exclusion criteria for the analysis.

**TABLE 1:** The demographic and clinical characteristics of all patients.

Characteristics	*N* (%)
Age, no. (%)	
< 60 years	83 (48.0)
≥ 60 years	90 (52.0)
Sex, no. (%)	
Male	124 (71.7)
Female	49 (28.3)
Tumor location, no. (%)	
Upper	52 (30.1)
Middle	47 (27.2)
Lower	64 (37.0)
Overlap	5 (2.9)
Unknown	5 (2.9)
Becker‐TRG, no. (%)	
1a	15 (8.7)
1b	21 (12.1)
2	56 (32.4)
3	81 (46.8)
cT, no. (%)	
T2	1 (0.6)
T3	36 (20.8)
T4a	120 (69.4)
T4b	16 (9.2)
cN, no. (%)	
N0	4 (2.3)
N+	169 (97.7)
cM, no. (%)	
M0	116 (67.1)
M1	57 (32.9)
Unresectable reason, no. (%)	
Locally unresectable	5 (2.9)
Retroperitoneal or distant region lymph nodes	15 (8.7)
Positive peritoneal lavage cytology	3 (1.7)
Peritoneal cavity metastasis	20 (11.6)
Distant organ metastasis	20 (11.6)
Histological type, no. (%)	
Adenocarcinoma	137 (79.2)
Signet ring cell carcinoma	36 (20.8)
Lauren type, no. (%)	
Intestinal	65 (37.6)
Diffuse	35 (20.2)
Mixed	35 (20.2)
Unknown	38 (22.0)
Histological grade, no. (%)	
Moderately and well differentiated	39 (22.5)
Poorly differentiated	78 (45.1)
Unknown	56 (32.3)
Preoperative treatment, no. (%)	
Neoadjuvant	105 (60.7)
Conversion	68 (39.3)
Chemotherapy regimes, no. (%)	
Nonplatinum	49 (38.8)
Platinum‐based	119 (52.9)
Treatment cycles in total, no. (%)	
≤ 4 cycles	125 (72.2)
> 4 cycles	48 (27.8)
Complete pathological response (pCR), no. (%)	
No	158 (91.3)
Yes	15 (8.7)

### 3.2 Association Between Clinicopathological Characteristics and pCR

To investigate prognostic factors associated with pCR. Patients and tumor characteristics were compared between pCR group and non‐pCR group, respectively (Table 2). There were no statistical differences between the two groups, including age, gender, tumor site, Lauren classification, differentiation, and preoperative chemotherapy. Patients with cM0 stage had a higher rate of pCR compared to those with cM1 stage (OR: 0.291, 95% CI: 0.098–0.863, *p* = 0.026).

**TABLE 2:** Correlations between clinicopathologic factors and pathological complete response.

	non‐pCR	pCR	*p* ^a^	OR	95% CI		*p* ^a^
	*N* = 158	15			Lower	Upper	
Clinical characteristics							
Age of diagnosis [median (IQR), yr]	59.1 [57.4, 60.8]	62.7 [56.5, 68.8]	0.234	1.034	0.979	1.093	0.234
Sex (%)							
Male	112 (70.8)	12 (80.0)	0.561	0.609	0.164	2.258	0.458
Female	46 (29.2)	3 (20.0)		Ref			
Tumor site (%)							
Upper1/3	51 (32.3)	3 (20.0)	0.174	Ref			
Middle1/3	47 (29.7)	2 (13.3)		0.324	0.084	1.242	0.100
Lower1/3	55 (34.8)	10 (66.7)		0.234	0.049	1.122	1.122
Unclear^b^	5 (3.2)	0 (0.0)					
cT stage (%)							
cT2	1 (0.7)	0 (0.0)	0.269	0	0		1
cT3	35 (22.1)	1 (6.7)		0.249	0.032	1.960	0.187
cT4	122 (77.2)	14 (93.3)		Ref			
cN stage (%)							
N0	4 (2.5)	0 (0.0)	0.999	0	0		0.999
N+	154 (78.5)	15 (100.0)		Ref			
cM stage (%)							
M0	110 (69.7)	6 (40.0)	**0.040**	0.291	0.098	0.863	**0.026**
M1	48 (30.3)	9 (60.0)		Ref			
Unresectable reason (%)							
Locally unresectable	5 (9.3)	0 (0.0)	0.221	0	0		0.999
Retroperitoneal or distant region lymph nodes	13 (24.1)	2 (22.2)		0.359	0.061	2.106	0.256
Positive peritoneal lavage cytology	3 (5.6)	0 (0.0)		0	0		0.999
Peritoneal cavity metastasis	19 (35.2)	1 (11.1)		0.123	0.013	1.128	0.065
Distant organ metastasis	14 (25.8)	6 (66.7)		Ref			
Signet‐ring cell carcinoma (%)							
No	123 (77.8)	14 (93.3)	0.200	3.984	0.506	31.357	0.189
Yes	35 (22.1)	1 (6.7)		Ref			
Lauren classification (%)							
Diffuse	39 (24.6)	4 (26.7)	**0.031**	4.308	0.461	40.231	0.2
Intestinal	72 (45.5)	7 (46.7)		4.083	0.485	34.346	0.195
Mixed	42 (26.5)	1 (6.7)		Ref			
Unclear^b^	5 (3.1)	3 (20.0)					
Stage of differentiation (%)							
G2	52 (32.9)	6 (40.0)	0.999	1.471	0.485	4.464	0.495
G3	102 (64.5)	8 (53.3)		Ref			
Unclear^b^	4 (2.5)	1 (6.7)					
Preoperative treatment							
Neoadjuvant	99 (62.6)	6 (40.0)	0.102	0.397	0.135	1.172	0.095
Conversion	59 (37.3)	9 (60.0)		Ref			
Laboratory characteristics							
B cell ratio [median (IQR), %]	10.4 [10.1, 11.4]	7.5 [5.4, 8.9]	**0.008**	0.702	0.562	0.877	**0.002**
T cell ratio [median (IQR), %]	70.5 [69.0, 72.0]	66.9 [60.8, 73.0]	0.175	0.968	0.923	1.015	0.177
Th cell ratio [median (IQR), %]	43.0 [41.6, 44.4]	41.4 [35.5, 47.3]	0.520	0.981	0.927	1.039	0.517
Ts cell ratio [median (IQR), %]	24.9 [23.5, 26.3]	23.1 [17.8, 28.4]	0.445	0.975	0.914	1.04	0.443
NK cell ratio [median (IQR), %]	17.7 [16.3, 19.3]	23.7 [20.3, 28.7]	**0.011**	1.069	1.019	1.121	**0.006**

*Note:* For continuous variables, ORs are reported per 1% increase for lymphocyte subset ratios and per 1‐year increase for age.

Abbreviations: pCR, pathological complete response; Ref, reference; yr., year.

^a^ Significant *p*‐values are indicated in bold.

^b^ Unclear and mixed are not included in analysis.

We further explored the relationships among pCR, peripheral lymphocyte subsets, and metastatic status by comparing pCR status and lymphocyte profiles between M0 and M1 subgroups. Although the M1 subgroup exhibited a higher proportion of pCR, no significant differences were identified in peripheral lymphocyte characteristics (OR: 0.291, 95% CI: 0.098–0.863, *p* = 0.026) (Table S1). Additionally, no statistically significant differences in lymphocyte subsets were observed between patients receiving neoadjuvant therapy and those undergoing conversion therapy (Table S2).

### 3.3 Predictive Value of Peripheral Lymphocyte Subsets for pCR in Gastric Cancer

Preoperative peripheral lymphocyte parameters were compared between pCR group and non‐pCR group, respectively. The ratio and absolute count of CD19^+^ B cells were significantly lower in the pCR group compared to the non‐pCR group (Figure 2A,B). Conversely, the ratio and absolute count of CD16^+^CD56^+^ NK cells were significantly higher in the pCR group than in the non‐pCR group (Figure 2C,D). No statistically significant differences were observed between the two groups for the ratios and counts of CD4^+^ T cells and CD8^+^ T cells (Figure 2E–H).

**FIGURE 2:**
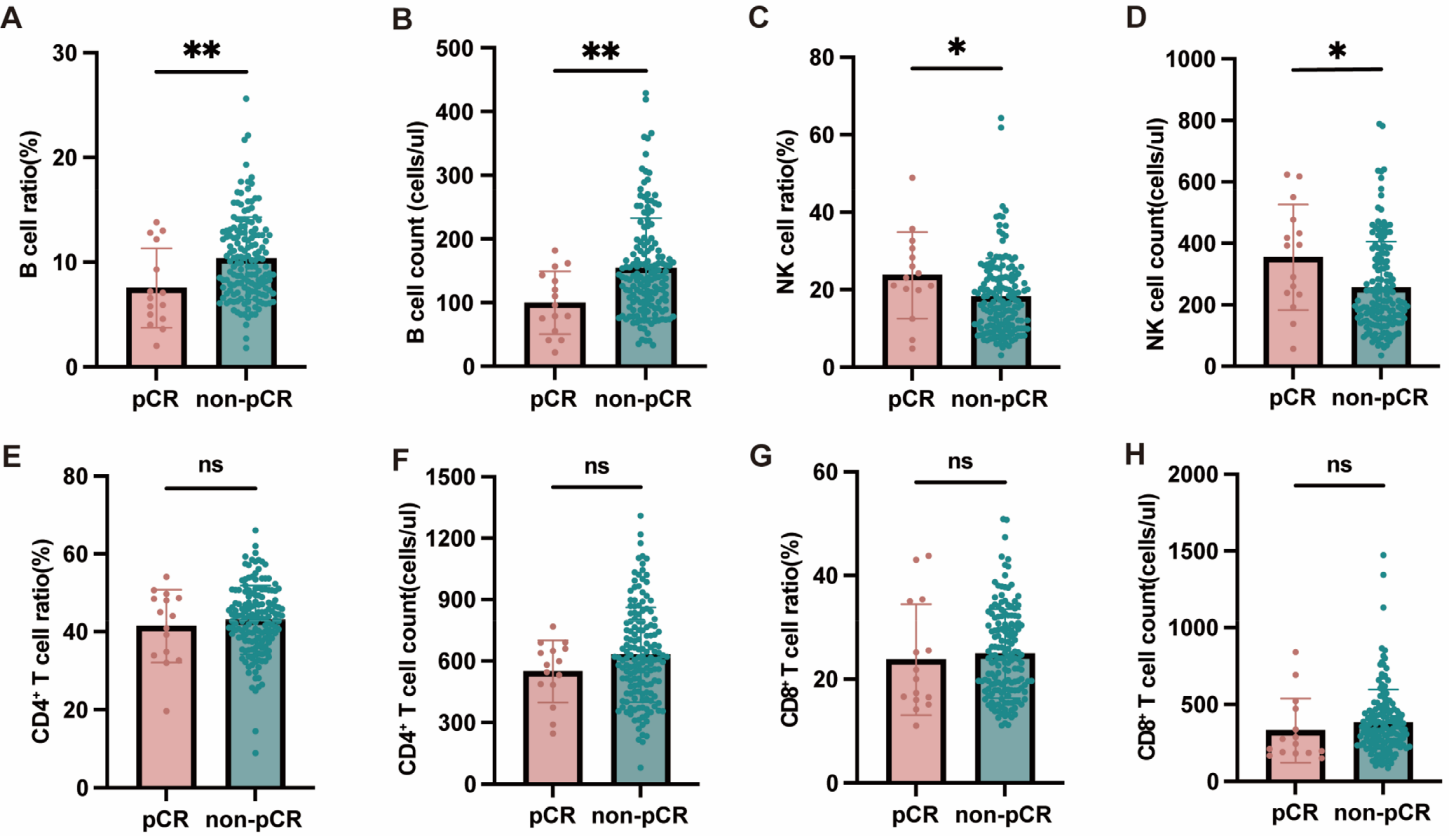
Correlation between preoperative peripheral lymphocyte subsets and pCR. pCR is correlated with lower B cell ratio and count (A and B). pCR is correlated with higher NK cell ratio and count (C and D). No significant differences were seen in CD4^+^ T cell and CD8^+^ T cell ratios and counts between pCR and non‐pCR group (E–H). *p < 0.05, **p < 0.01, ns: not significant, *t*‐test.

To further elucidate the prognostic significance of these parameters, multivariate analysis confirmed that a higher B cell ratio was independently associated with poorer outcomes (Table 3).

**TABLE 3:** Multivariate analysis: factors associated with pathological complete response.

	95% CI			
	OR	Lower	Upper	*p* ^a^
Age of diagnosis [median (IQR), yr]	1.001	0.942	1.084	0.766
cM stage (%)				
M0	0.308	0.080	1.185	0.087
M+	1			
Lauren classification (%)				
Diffuse	2.896	0.266	31.484	0.382
Intestinal	3.562	0.391	32.451	0.260
Mixed	1			
B cell ratio [median (IQR), %]	0.751	0.592	0.953	**0.018**
NK cell ratio [median (IQR), %]	1.066	0.998	1.139	0.057

^a^ Significant *p*‐values are indicated in bold.

To ascertain the best predictive value of peripheral lymphocyte subsets, ROC curve analysis was performed. For the B cell ratio, the optimal cut‐off was 7.250 with a sensitivity of 75.95% and a specificity of 66.67% in predicting the efficacy of preoperative chemotherapy leading to pCR (Figure S2A). Collectively, these findings underscore the predictive significance of preoperative peripheral B cells in response to chemotherapy.

### 3.4 Prognostic Impact of Peripheral Lymphocyte Subsets on Survival in Gastric Cancer

Patients with a high NK cell count (> 250 cells/μL) exhibited superior OS compared to those with a low NK cell count (≤ 250 cells/μL), with a statistically significant difference (HR (95% CI) = 1.991 (1.026–3.862), *p* = 0.044) (Figure 3A). Other markers such as B cell count (> 144 cells/μL vs. ≤ 144 cells/μL, HR (95% CI) = 0.7912 (0.4068–1.539), *p* = 0.480), CD4^+^ T cell count (> 639 cells/μL vs. ≤ 639 cells/μL, HR (95% CI) = 1.252 (0.6452–2.429), *p* = 0.500) and CD8^+^ T cell count (> 367 cells/μL vs. ≤ 367 cells/μL, HR (95% CI) = 0.7478 (0.3851–1.452), *p* = 0.390) were also assessed for their association with OS, but none were found to be significantly correlated (Figure 3B–D). While multivariate Cox regression analysis did not reach statistical significance (*p* = 0.074), the HR (95% CI) = 2.033 (0.933–4.43) remained consistent in direction with the univariate analysis (Figure 4). This suggested that a preoperative high peripheral NK cell count is correlated with improved survival in univariate analysis.

**FIGURE 3:**
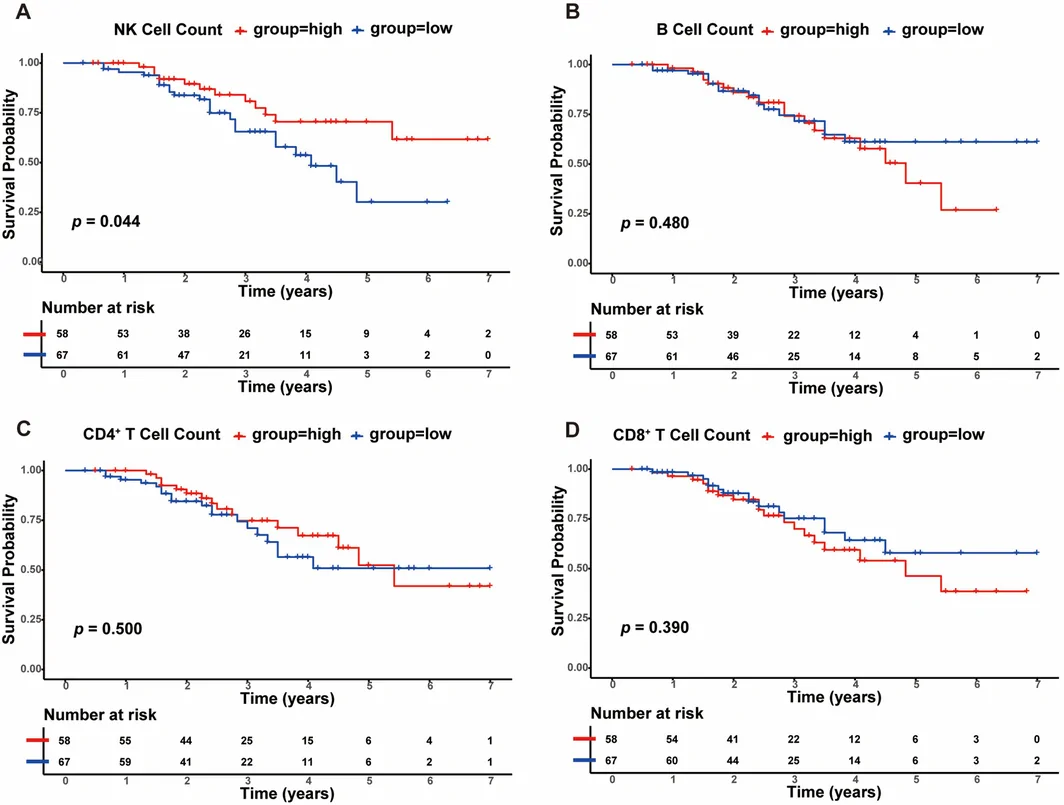
Overall survival of patients with gastric cancer based on peripheral lymphocyte subsets. High‐NK cell count group was associated with better OS (HR (95% CI) = 1.991 (1.026–3.862), *p* = 0.044) (A). B cell count (HR (95% CI) = 0.7912 (0.4068–1.539), *p* = 0.480) (B), CD4^+^ T cell count (HR (95% CI) = 1.252 (0.6452–2.429), *p* = 0.500) (C), and CD8^+^ T cell count (HR (95% CI) = 0.7478 (0.3851–1.452), *p* = 0.390) (D) were not significantly associated with OS.

**FIGURE 4:**
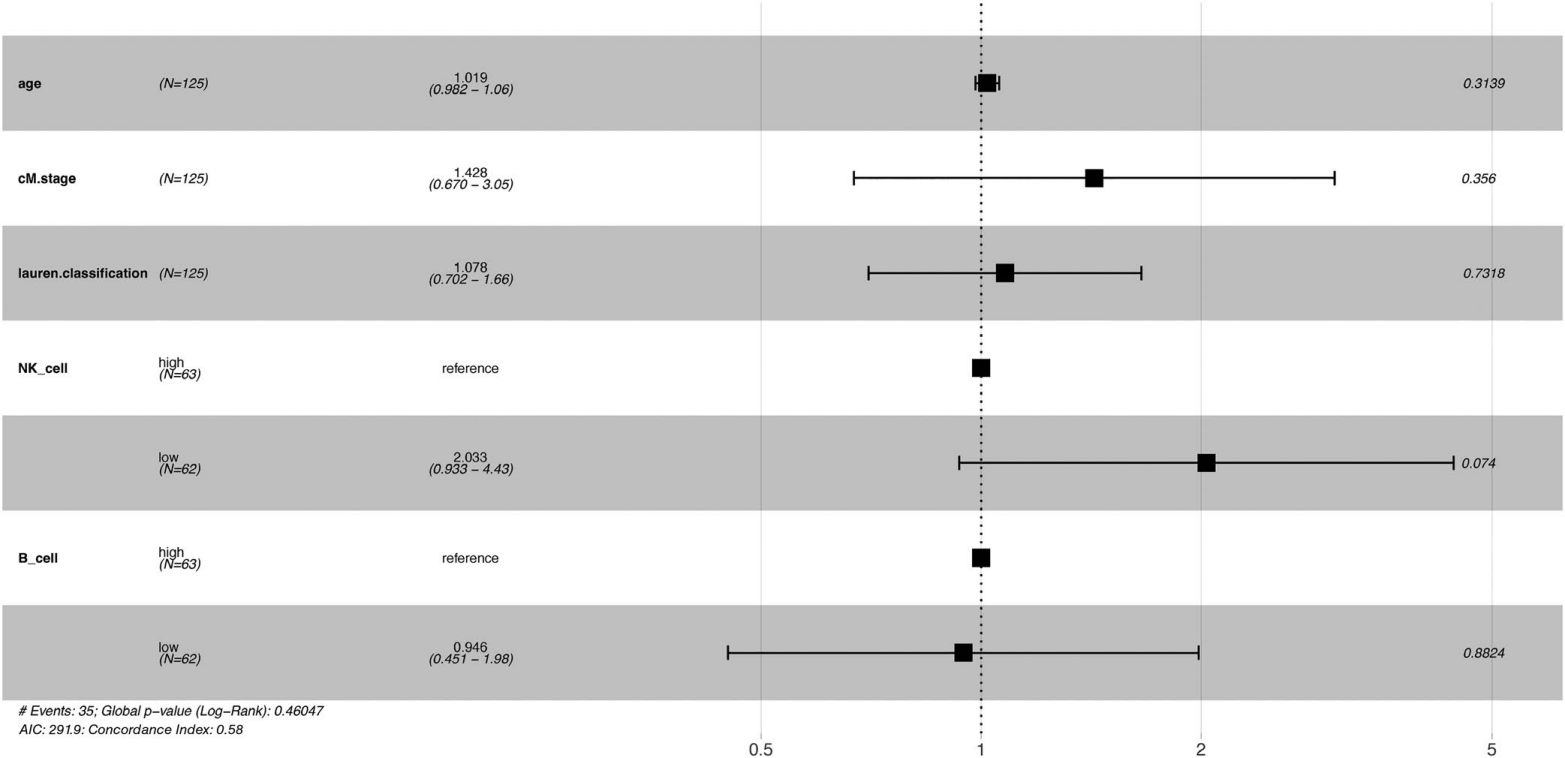
Forest plot of multivariate Cox regression analysis for overall survival. The model was adjusted for age, gender, TNM stage, and treatment. Among all variables, NK cell abundance was not identified as an independent prognostic factor (HR (95% CI) = 2.033 (0.933–4.43), *p* = 0.074). *p < 0.05. OS, overall survival. CI, confidence interval; HR, hazard ratio.

### 3.5 Associations Between Preoperative Peripheral Lymphocyte Subsets and Cytokines

To investigate the correlation between the proportion of peripheral lymphocyte subsets and treatment response, we accessed the cytokine levels, including TNF, IL‐8, sIL‐2R and IL‐6 with preoperative peripheral subsets. Elevated levels of TNF (29.4 pg/mL vs. 11.8 pg/mL, *p* = 0.005) and IL‐8 (42.3 pg/mL vs. 20.6 pg/mL, *p* = 0.005) after the initial chemotherapy cycle were associated with better treatment response. However, no significant differences were observed between the two groups for sIL‐2R (548.1 U/mL vs. 439.8 U/mL, *p* = 0.071) and IL‐6 (6.5 pg/mL vs. 5.5 pg/mL, *p* = 0.522) (Figure 5A–D). Spearman correlation analysis was performed to evaluate the associations between the B cell ratio and NK cell ratio and the levels of cytokines, and B‐cell absolute count was weakly negatively correlated with tumor necrosis factor (TNF; Spearman's *ρ* = −0.307, *p* = 0.035). No significant correlations were observed between B cell absolute count and sIL‐2R or IL‐8, or between NK cell ratio and any of the cytokines.

**FIGURE 5:**
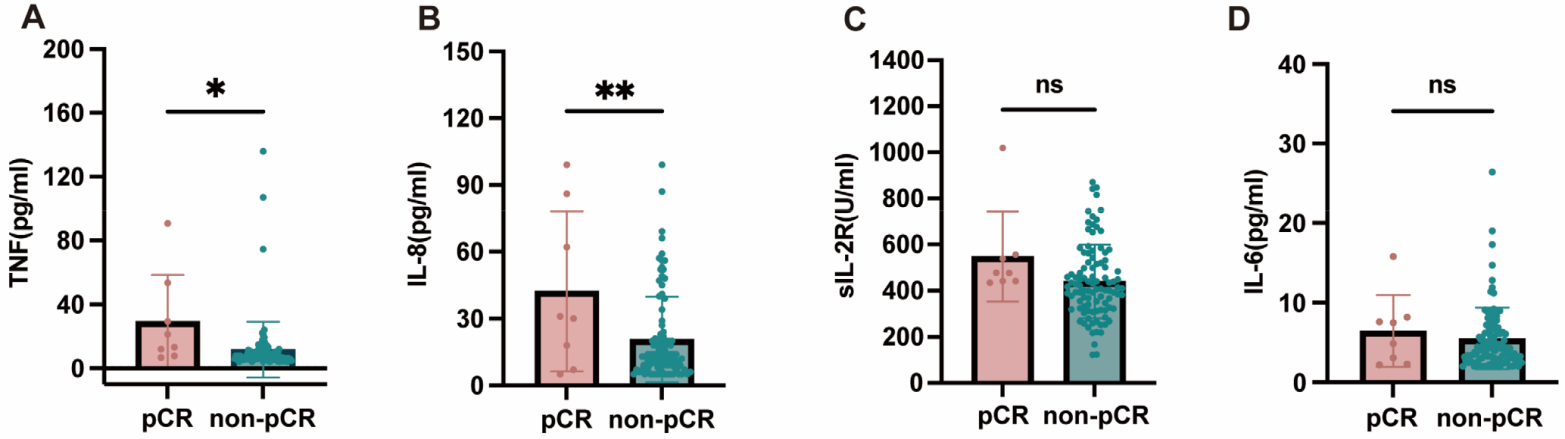
The correlation between peripheral lymphocyte subsets and the characteristics of peripheral pro‐inflammatory cytokines among patients with gastric cancer. pCR was correlated with higher concentration of TNF and IL‐8 (A and B). sIL‐2R and IL‐6 secretion were not significantly associated with pCR (C and D). *p < 0.05, **p < 0.01, ns: not significant, *t*‐test. IL‐6, interleukin‐6; IL‐8, interleukin‐8; sIL‐2R, soluble interleukin‐2 receptor; TNF, tumor necrosis factor.

### 3.6 Associations Between Peripheral Lymphocyte Subsets and Tumor‐infiltrating Lymphocytes in the TME

Considering the correlation between local tumor immune responses and systemic immune responses, as well as the role of TILs in tumor progression and clinical treatment response [25], we proceeded to explore the correlation between tumor‐infiltrating lymphocyte subsets and OS in gastric cancer. To validate the concordance between peripheral lymphocyte subsets and TILs, CIBERSORT analysis was conducted to identify the characteristic of TIL based on B cells (CD19^+^, MS4A1^+^) infiltration of the Yonsei cohort. In line with the patterns observed in peripheral lymphocyte subsets, an increase in NK cells (NCR1^+^, FGFBP2^+^) was observed in the B cell low group (Figure 6A). Although no significant difference was seen between B cell high/low subgroups (Figure 6B).

**FIGURE 6:**
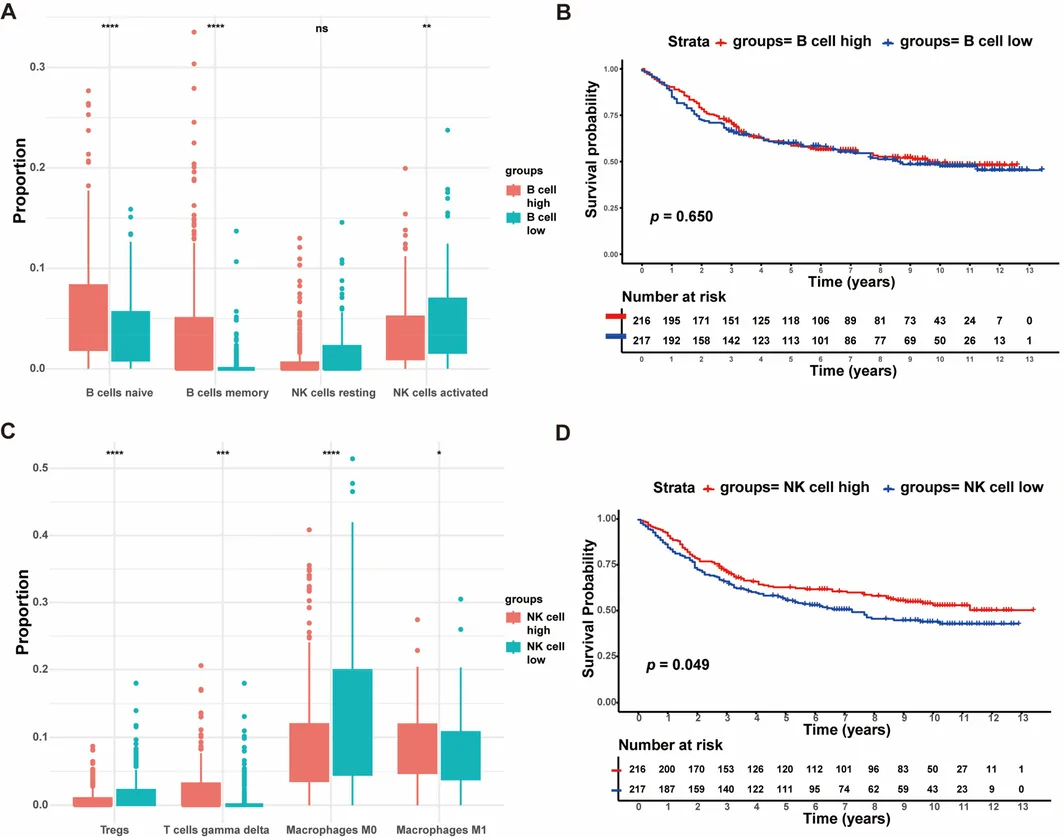
Identification of immunoevasive contexture based on NK cell count in gastric cancer. B cells showed a significantly inverse correlation with NK cells in the tumor microenvironment (TME) (A). B cell infiltration did not influence the OS in the TME (B). NK cells are positively correlated with T cells gamma delta and Macrophages M1 and negatively correlated with Tregs and Macrophages M1 (C). High NK cell infiltration predicted significantly better survival outcomes in the TME (D). *p < 0.05, **p < 0.01, ***p < 0.001, ****p < 0.0001, ns: not significant, *t*‐test.

We further analyzed the infiltration of other TILs in NK cell high/low tumors using CIBERSORT analysis. Increased Macrophages M0 and Tregs were observed in the low NK cell group, and decreased Macrophages M1 and T cells gamma delta were observed in the low NK cell group, correlating with poor prognosis (Figure 6C).

The NK cell high group also showed a correlation with improved OS (*p* = 0.049) (Figure 6D). Consequently, these findings implied that reduced NK cell infiltration may be associated with an immunosuppressive TME phenotype in gastric cancer.

## 4 Discussion

Cancer is a complex disease, with both systemic immune response and the local TME playing crucial roles in determining treatment outcomes [18]. Peripheral lymphocytes are instrumental in tumor‐associated inflammation and immune responses [26]. In this retrospective study, we evaluated the correlation between peripheral lymphocyte subsets and pCR in patients with gastric cancer. Our findings indicated that higher proportions and absolute counts of NK cells, together with lower proportions and absolute counts of B cells in peripheral blood, were associated with a more favorable response to preoperative chemotherapy. Analysis of peripheral blood prior to curative gastrectomy provides a practical, noninvasive, and cost‐effective method to assess tumor response to treatment, offering critical insights into selecting patients likely to benefit from preoperative chemotherapy.

The relationship between pCR and OS remains controversial. While some studies have reported that pCR of the primary tumor (ypT0) after preoperative therapy correlates with improved OS [8, 27, 28], others have found no such association [29, 30]. Thus, the prognostic value of pCR for OS remains inconclusive. It is possible that pCR affects long‐term survival indirectly through immune‐related mechanisms. In our study, no direct correlation was observed between pCR and OS, though the limited number of pCR cases may have introduced bias.

Conventional cancer therapies, including chemotherapy and surgery, can significantly perturb the global immune landscape. Peripheral lymphocyte subsets, which partially reflect the systemic immune status of patients, have been proved to be valuable prognostic factors in various malignancies [31, 32]. Systemic immunology markers such as NLR, PLR, SII have been identified as predictors of clinical efficacy [33, 34, 35]. Wu, et al. found that clonotypic expansion of effector‐like T cells in the peripheral blood can represent the overall strength of immune response and predict the immunotherapy efficacy [36]. Kobayashi et al. emphasized the importance of cytotoxic CD8^+^ T cells in suppressing tumor growth and CD4^+^ T cells in orchestrating immune responses in lung cancer [37]. Similarly, peripheral CD3^+^ T cells, CD8^+^ T cells, and NK cells have been reported to have prognostic value in breast cancer [38, 39, 40]. Our study pointed out that a low proportion of NK cells before surgery may indicate impaired immune function, negatively affecting the preoperative chemotherapy prognosis of gastric cancer patients.

NK cells are crucial components of antitumor immunity, primarily acting within the innate immune system. Studies on colorectal cancer and nonsmall‐cell lung cancer showed that a decreased peripheral NK cell count is an independent risk factor for poor prognosis [41, 42]. NK cells eliminate tumor cells through multiple mechanisms, including antibody‐dependent cellular cytotoxicity, direct cytolytic activity, and cytokine‐mediated killing. Our findings revealed that a high absolute count of preoperative peripheral NK cells was associated with favorable OS in univariable analysis. This prognostic association was attenuated in the multivariable model, suggesting that the effect of NK cell abundance may be partly mediated by other clinical covariates, such as tumor burden or systemic inflammation, and that the analysis may have been underpowered; validation in larger prospective cohorts is therefore required. Nevertheless, the univariable association indicates that peripheral NK cell levels reflect the host's baseline antitumor immune competence and may serve as a useful parameter for preliminary patient stratification. Considered alongside the analysis of the Yonsei cohort, these findings suggest that the peripheral blood immune profile may partly mirror immune cell densities within the TME. The interplay between peripheral immunity and the immunosuppressive TME is complex. Because peripheral lymphocyte subsets can be evaluated readily in blood samples, invasive procedures such as tissue biopsy may be partially avoidable when assessing the tumor immune microenvironment. These findings underscore the potential of peripheral NK cells as both prognostic indicators and therapeutic targets in gastric cancer immunotherapy.

B cells are traditionally recognized for their role in positively modulating immune responses and inflammation through antibody production, as well as promoting T‐cell activation and proliferation via antigen presentation [43]. In gastric cancer, CD19^+^ regulatory B cells (Bregs) modulate the conversion of CD4^+^ T cells to Tregs by secreting IL‐10 and TGF‐β, and they can also impair the antitumor effect of CD4^+^ T cells by inhibiting IFN‐γ and TNF‐α [44]. Our study found that B cell ratio was weakly negatively correlated with TNF secretion, suggesting their potential immunosuppressive role in the TME. Shen et al. previously demonstrated that Bregs in gastric cancer suppress the production of cytokines like IFN‐γ and TNF‐α by CD4^+^ T cells, thereby dampening the immune response [45]. In line with this, the high proportion of CD19^+^ B cells observed in non‐pCR groups in our study may represent either Bregs or immature B cells. These findings suggest that an elevated proportion of CD19^+^ B cells, particularly those with regulatory or immature phenotypes, may act as critical mediators of immune suppression in gastric cancer and could serve as potential biomarkers or therapeutic targets.

This study has several limitations. The cohort included only 15 pCR and 158 non‐pCR patients, increasing the risk of sampling bias. The data on mature subsets of B cells and NK cells were incomplete; further exploration of specific B cell subtypes is necessary. We did not evaluate the phenotypes and functions of B cells and NK cells. The relationship between B cells and cytokines was not well understood, and the multifactorial analyses may introduce confounding variables. Further research at the RNA and protein levels is needed to validate their relationship and elucidate the specific mechanisms. Additionally, we did not analyze variables such as radiotherapy, hyperthermic intraperitoneal preoperative chemotherapy, immunotherapy, and radiotherapy in this study, and heterogeneous treatment regimens may also confound immune biomarker associations. Finally, the cut‐off values for peripheral lymphocyte subsets could differ based on various methods, potentially influencing the results.

In conclusion, this retrospective study revealed that a lower proportion of CD19^+^ B cells may serve as a predictive biomarker for pCR, while an increased peripheral NK cell count could function as an auxiliary factor for risk stratification when combined with clinical parameters for assessing long‐term outcomes in gastric cancer patients receiving preoperative chemotherapy. This study suggests that the preoperative chemotherapy efficacy could potentially be assessed noninvasively using peripheral lymphocyte subset markers.

## Author Contributions

**Zirui Xue:** formal analysis, writing – original draft, conceptualization, methodology, data curation, resources. **Dan Liu:** formal analysis, writing – original draft, conceptualization, methodology, resources. **Zhaodong Sun:** conceptualization, formal analysis, writing – original draft, methodology, resources. **Bosen Li:** visualization, software. **Xinyou Liu:** visualization, software. **Junjie Zhao:** visualization, software. **Haojie Li:** visualization, software. **Xuefei Wang:** investigation, supervision, project administration, writing – review and editing, validation, funding acquisition. **Zhaoming Wang:** writing – review and editing, project administration, investigation, supervision, validation, funding acquisition.

## Funding

This work was supported by Project of Science and Technology of Xiamen City (3502Z20224015) and National Youth Science Fund Project of National Natural Science Foundation of China (82403871).

## Ethics Statement

Ethical approval for this study (B2023‐229) was provided by the Ethical Committee of Zhongshan Hospital, Fudan University on 15 July 2023.

## Consent

Participants consent to publish their data.

## Conflicts of Interest

The authors declare no conflicts of interest.

## Supporting information


**Figure S1:** Overall survival of patients with or without pathological complete response (pCR).
**Figure S2:** Predictive value of preoperative peripheral lymphocyte subsets in patients with gastric cancer.
**Figure S3:** Gating strategy of preoperative peripheral lymphocyte subsets in patients with gastric cancer.

**Table S1:** Correlations between clinicopathologic factors and metastasis pattern.
**Table S2:** Correlations between clinicopathologic factors and treatment intent.

## Data Availability

Research data are not shared.
