# Supplementary material for: Peripheral Lymphocyte Subsets Predict Efficacy and Overall Survival in Gastric Cancer Patients Receiving Preoperative Chemotherapy: A Retrospective Cohort Study
Source: Cancer Rep (Hoboken). 2026 Oct 13;9(10):e70704. doi: 10.1002/cnr2.70704 (PMC13659800; doi:10.1002/cnr2.70704)
Supplement: Supplementary file 1 [file CNR2-9-e70704-s001.docx]

**Supplement Table**

**Table S1.** Correlations between clinicopathologic factors and metastasis pattern.

|  | M0 | M1 |  |  | 95%CI | |  |
| --- | --- | --- | --- | --- | --- | --- | --- |
|  | N=116 | 57 | P^a^ | OR | lower | upper | P^a^ |
| Pathological response | | | | | | | |
| pCR (%) |  |  |  |  |  |  |  |
| No | 110 (94.8) | 48 (84.2) | 0.040 | 0.291 | 0.098 | 0.863 | 0.026 |
| Yes | 6 (5.1) | 9 (15.7) |  | 1.000 |  |  |  |
| Preoperative peripheral markers | | | | | | | |
| B cell ratio [median (IQR), %] | 10.6 [9.8,11.3] | 10.1 [9.0,11.1] | 0.430 | 0.968 | 0.894 | 1.049 | 0.428 |
| T cell ratio [median (IQR), %] | 70.4 [68.7,72.2] | 69.7 [66.9,72.5] | 0.648 | 0.993 | 0.961 | 1.025 | 0.646 |
| Th cell ratio [median (IQR), %] | 43.0 [41.4,44.7] | 42.5 [40.1,45.0] | 0.740 | 0.981 | 0.927 | 1.039 | 0.738 |
| Ts cell ratio [median (IQR), %] | 25.2 [23.6,26.9] | 23.8 [21.7,26.0] | 0.311 | 0.981 | 0.945 | 1.018 | 0.310 |
| NK cell ratio [median (IQR), %] | 17.8 [16.3,19.3] | 19.3 [16.7,21.8] | 0.458 | 1.012 | 0.980 | 1.046 | 0.456 |

^a^Significant P-values are indicated in bold.

^b^Unclear and mixed are not included in analysis.

pCR, pathological complete response; yr, year.

**Table S2.** Correlations between clinicopathologic factors and treatment intent.

|  | Neoadjuvant therapy | | Conversion therapy |  |  | 95%CI | |  |
| --- | --- | --- | --- | --- | --- | --- | --- | --- |
|  | N=105 | | 68 | P^a^ | OR | lower | upper | P^a^ |
| Pathological response | | | | | | | | |
| pCR (%) |  | |  |  |  |  |  |  |
| No | 99 (94.2) | | 59 (86.7) | 0.097 | 0.397 | 0.135 | 1.172 | 0.095 |
| Yes | 6 (5.7) | | 9 (13.2) |  | 1.000 |  |  |  |
| Preoperative peripheral markers | | | | | | | | |
| B cell ratio [median (IQR), %] | | 10.8 [10.0,11.6] | 9.8 [8.9,11.1] | 0.130 | 0.942 | 0.871 | 1.018 | 0.132 |
| T cell ratio [median (IQR), %] | | 70.7 [68.9,72.6] | 69.3 [66.8,71.8] | 0.358 | 0.986 | 0.956 | 1.017 | 0.358 |
| Th cell ratio [median (IQR), %] | | 43.2 [41.4,44.9] | 42.4 [40.2,44.5] | 0.562 | 0.990 | 0.957 | 1.024 | 0.560 |
| Ts cell ratio [median (IQR), %] | | 25.4 [23.6,27.1] | 23.8 [21.9,25.7] | 0.241 | 0.979 | 0.944 | 1.015 | 0.241 |
| NK cell ratio [median (IQR), %] | | 17.6 [15.7,19.4] | 19.8 [17.5,22.2] | 0.126 | 1.025 | 0.993 | 1.058 | 0.128 |

^a^Significant P-values are indicated in bold.

^b^Unclear and mixed are not included in analysis.

pCR, pathological complete response; yr, year.
