# Supplementary material for: Peripheral Lymphocyte Subsets Predict Efficacy and Overall Survival in Gastric Cancer Patients Receiving Preoperative Chemotherapy: A Retrospective Cohort Study
Source: Cancer Rep (Hoboken). 2026 Oct 13;9(10):e70704. doi: 10.1002/cnr2.70704 (PMC13659800; doi:10.1002/cnr2.70704)
Supplement: Supplementary file 2 [file CNR2-9-e70704-s002.docx]

**Supplement Figure**

**Figure S1.** Overall survival of patients with or without pathological complete response (pCR).

**Figure S2.** Predictive value of preoperative peripheral lymphocyte subsets in patients with gastric cancer. B cell ratio (A), NK cell ratio (B), B cell count (C), and NK cell count (D) all suggested good capacity for the identification of pCR.

**Figure S3.** Gating strategy of preoperative peripheral lymphocyte subsets in patients with gastric cancer.
